# Prize Essay

**Published:** 1856-11

**Authors:** 


*Prize Essay.*

At the last annual meeting of the "Illinois" State Medical Society, the sum of *Fifty Dollars* was provided and offered as a premium for the best Essay on some Medical subject; and we hope it will not be forgotten by the profession.

All essays, intended as competitors for this premium, should be transmitted to Prof. J. V. Z. Blaney, Chairman of the Committee appointed to examine the same, on or before the *first* day of May, 1857.

Each essay should have a motto and be accompanied by a sealed note with the same motto on the outside, and within the name of the author.

